# Lignin from Hardwood and Softwood Biomass as a Lubricating Additive to Ethylene Glycol

**DOI:** 10.3390/molecules23030537

**Published:** 2018-02-28

**Authors:** Liwen Mu, Jian Wu, Leonidas Matsakas, Minjiao Chen, Alireza Vahidi, Mattias Grahn, Ulrika Rova, Paul Christakopoulos, Jiahua Zhu, Yijun Shi

**Affiliations:** 1 Division of Machine Elements, Luleå University of Technology, 97187 Luleå, Sweden; fujin.1234@163.com (L.M.); 790532909@163.com (J.W.); mjchen.yzu@gmail.com (M.C.); alirezavahidi@hotmail.com (A.V.); 2 Intelligent Composites Laboratory, Department of Chemical and Biomolecular Engineering, The University of Akron, Akron, OH 44325, USA; 3 Biochemical Process Engineering, Division of Chemical Engineering, Department of Civil, Environmental and Natural Resources Engineering, Luleå University of Technology, 97187 Luleå, Sweden; leonidas.matsakas@ltu.se (L.M.); Ulrika.Rova@ltu.se (U.R.); paul.christakopoulos@ltu.se (P.C.); 4 Chemical Technology, Division of Chemical Engineering, Department of Civil, Environmental and Natural Resources Engineering, Luleå University of Technology, 97187 Luleå, Sweden; Mattias.Grahn@ltu.se

**Keywords:** lignin, organosolv, lubrication, biomass, hydrogen bonding

## Abstract

Ethylene glycol (EG)-based lubricant was prepared with dissolved organosolv lignin from birch wood (BL) and softwood (SL) biomass. The effects of different lignin types on the rheological, thermal, and tribological properties of the lignin/EG lubricants were comprehensively investigated by various characterization techniques. Dissolving organosolv lignin in EG results in outstanding lubricating properties. Specifically, the wear volume of the disc by EG-44BL is only 8.9% of that lubricated by pure EG. The enhanced anti-wear property of the EG/lignin system could be attributed to the formation of a robust lubrication film and the strong adhesion of the lubricant on the contacting metal surface due to the presence of a dense hydrogen bonding (H-bonding) network. The lubricating performance of EG-BL outperforms EG-SL, which could be attributed to the denser H-bonding sites in BL and its broader molecular weight distribution. The disc wear loss of EG-44BL is only 45.7% of that lubricated by EG-44SL. Overall, H-bonding is the major contributor to the different tribological properties of BL and SL in EG-based lubricants.

## 1. Introduction

Lignin, a cross-linked polymer with phenylpropanoic monomers, is the second most abundant biopolymer in nature [1]. It holds a great potential to be converted to high value-added phenolic platform chemicals taking advantage of its abundant phenolic structure [2]. Currently, 70 million tons of lignin are produced annually worldwide, yet only 5% is effectively converted to valuable chemicals, while the remaining 95% is primarily burnt to recover energy in the pulp and paper industry [3,4]. Effective depolymerization of lignin is a great challenge that needs to be overcome in order to utilize lignin as a high value-added renewable resource available in massive quantities [5].

In addition, the structure and chemical composition of lignin vary in different plant species, or while using different extraction processes and subsequent treatments, thus increasing the complexity of lignin processing and decreasing its applicability in industrial processes [6,7]. In general, the following methods have been used to extract lignin from lignocellulosic feedstock: an enzymatic process, the Klason method, the Kraft process, the organosolv process, and through ionic liquid (IL) selective pretreatments [8,9,10]. The Kraft lignin process uses sodium sulfide in an alkaline aqueous solution as a reaction medium to cleave the ester bonds between hemicellulose and lignin macromolecules [7]. The Klason method uses strong mineral acids (such as sulfuric acid) to remove the carbohydrate portion from lignocellulosic biomass, leaving lignin as an insoluble residue with a high lignin yield [11,12]. These two methods produce a large amount of wastewater during the isolation process and cause irreversible reactions that severely change the structure of the isolated lignin. In contrast, the organosolv process uses organic solvents to extract lignin from a biomass feedstock under mild conditions, which has a light impact on the environment [13].

**Figure 1:**
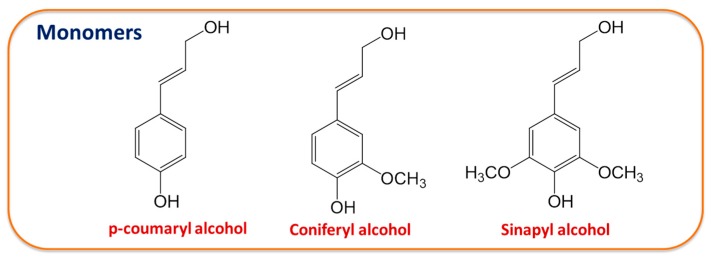
The three monomer building blocks of lignin.

According to the literature to date, lignin could be isolated from a wide range of plant biomass resources including but not limited to wood, grass, and bamboo [14,15,16]. The structures and physicochemical properties of lignin are related to plant taxonomy. For example, softwood lignin contains more coniferyl alcohol monolignol, whereas hardwood lignin features a mixture of coniferyl alcohol and sinapyl alcohol monolignol, and grass lignin presents a mixture of all three aromatic units (*p*-coumaryl alcohol, coniferyl alcohol, and sinapyl alcohol), as shown in Figure 1 [17]. Bai et al. [18] investigated the effect of different biomass species on the catalytic pyrolysis of lignin and found that herbaceous biomass lignin has the highest potential for pyrolytic conversion due to its highly branched polymer structure enriched in tricin, ferulate, and coumarate groups. 

Lubricants have been essential to human activity, with their purpose gradually shifting from “mobility” in ancient eras to “durability” and “energy efficiency” in modern times [19]. Considering the working principles of lubricants, the majority of lubricants rely on two factors: one is the adhesion of lubricants on a friction surface, and the other is the mechanical strength of a lubrication film [20,21]. Enhanced surface adhesion could be achieved by introducing hydrogen bonding (H-bonding), polar groups, and negative charges into the lubricant additive [22,23]. For example, the abundant carboxylic acid groups in a gelatin molecular structure are able to form a strong bond with hydrophilic surfaces [24]. An H-bonding network of phosphoric acid and water molecules adsorbed on sapphire and ruby surfaces could lead to an ultra-low friction coefficient [25]. Lubricants consisting of alcohols show a friction-reducing effect because an alcohol undergoes H-bonding with the oxide-rich metal surface [26]. Strengthening the interactions between the lubricant base and an additive through internal H-bonding seems to be another effective approach to improve the overall lubrication performance [27,28]. For example, the dispersion of reduced graphene oxide (rGO) in poly(ethylene glycol) 200 was improved via the H-bonding between the hydroxyl groups of rGO and the oxygen atoms of PEG200 molecules, thereby reducing the friction coefficient [29,30,31]. 

Recently, lignin was demonstrated as an effective lubricant additive in ionic liquids [20], base oil [27,32], and cutting fluids (metalworking fluids) [33] to reduce the friction coefficient and wear loss of metal/metal contacts due to the presence of effective H-bonding between lignin and the lubricant base. The H-bonding strength between lignin and the lubricant base could be modulated by the versatile molecular structures of lignin extracted from different plant species. However, to the best of our knowledge, the effect of lignin type on its efficiency as a lubricant additive has never been studied. In this work, ethylene glycol (EG) is selected as a base oil. Organosolv lignins isolated from birch (hardwood, BL) and spruce (softwood, SL) are used as additives to promote the lubricating efficiency of EG. The thermal, rheological, and tribological properties of the prepared EG-BL and EG-SL lubricants are systematically studied. The H-bonding between different lignin and EG and the effect of lignin molecular weight distribution on the lubricating performance of these new lubricants are investigated.

## 2. Results and Discussion

The average molecular weight of lignin is an important performance index when lignin is used as a lubricant additive. Table 1 shows the average molecular weight and polydispersity index (PDI) of organosolv lignin extracted from birch and spruce wood. SL has a larger *M_w_* and *M_n_* than that of BL. In addition, the PDI of BL is larger than that of SL, which indicates that BL has a broader molecular weight distribution.

Lignin mainly consists of three different cinnamyl alcohol monomers, i.e., *p*-coumaryl alcohol, coniferyl alcohol, and sinapyl alcohol [8,34]. The proportions of each monomer differ depending on the basis of lignin plant sources. SL in this work mainly consists of coniferyl alcohol units, whereas BL consists of both coniferyl and sinapyl alcohols units [17]. Concerning the molecular structure, there is one additional methoxy group in the sinapyl alcohol monomer as compared to the coniferyl alcohol monomer, which suggests that BL has more active sites to form H-bonding than SL.

**Figure 2:**
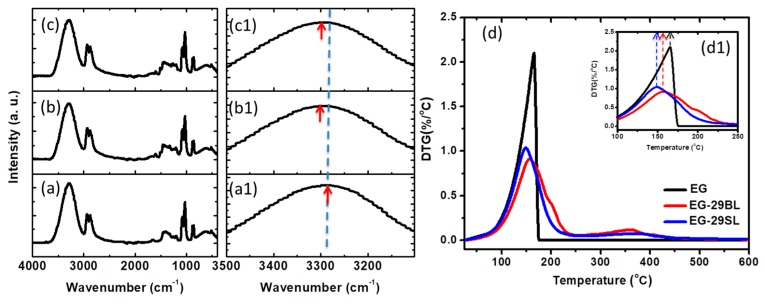
IR spectra of (**a**) ethylene glycol (EG); (**b**) EG-29BL; and (**c**) EG-29SL. Derivative thermogravimetric (DTG) curves of EG and EG/lignin under N_2_ atmosphere (**d**). (**a1**–**c1**) represent the enlarged IR spectra within the range of 3100–3500 cm^−1^; (**d1**) represents the enlarged DTG curve within the range of 100–250 °C.

The IR spectra in Figure 2a–c were used to characterize the lignin-EG interactions after microwave processing. EG in Figure 2a shows major absorption bands at 3200–3500, 2935/2877, and 1083/1032 cm^−1^, representing the O–H, C–H, and C–O stretching bands [35]. This shift in the OH wavenumber is an indicator of the change in intermolecular interactions [36,37]. It can be seen from Figure 2(a1–c1) that the O–H peak of the EG/lignin lubricant shifts to a higher wavenumber as compared with pure EG, which implies a weakened H-bonding after the addition of lignin. Lignin molecules are rich in proton donating groups (−OH) and proton accepting groups (−O− group), as seen in Figure 1. These groups will definitely interfere and break down the well-patterned H-bonding network in pure EG, thus decreasing the H-bonding density in the EG/lignin system [27]. The O–H peaks in EG-29BL and EG-29SL appeared at the same peak position, indicating that IR could not differentiate the interaction mode between different types of lignin and EG. 

The thermal stabilities of EG-based lubricants were further characterized. Figure 2d presents the derivative thermogravimetric (DTG) curves of EG-based lubricants. The addition of lignin in EG shifts the main degradation peak to a lower temperature. As observed in the IR results, the addition of lignin decreases the H-bonding density in the EG/lignin system, and this leads to a lower thermal degradation temperature. In addition, the thermal decomposition temperature of EG-BL is higher than that of EG-SL, indicating a stronger BL-EG interaction than that of SL-EG.

**Figure 3:**
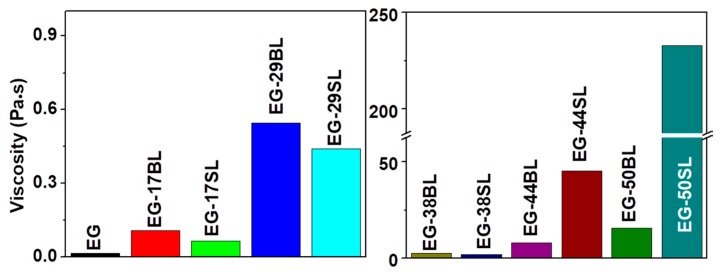
Viscosity of EG-based lubricants.

Since viscosity is closely related to the tribological properties of lubricants, it was measured and is summarized in Figure 3. Generally, the viscosity increases after dissolving lignin in EG and continuously increases with the increasing mass fraction of lignin. It is observed that the viscosity of EG-BL is larger than that of EG-SL at a lower lignin loading of less than 38%; this trend reverses at 44 and 50%. Such a difference in viscosity is determined by the thickening effect of lignin itself and the H-bonding interaction of EG/lignin. Enhanced H-bonding in the system is helpful to increase the viscosity. When the lignin loading is lower than 38%, the viscosity of EG-BL is higher than that of EG-SL, which could be attributed to a stronger H-bonding between BL and EG. The thickening effect of the polymer itself is more obvious with the increasing polymer concentration [38,39]. At 44% and 50%, the thickening effect becomes the dominating factor governing the viscosity. The viscosity of EG-BL is lower than that of EG-SL at 44% or 50% due to the higher molecular weight of SL. 

**Figure 4:**
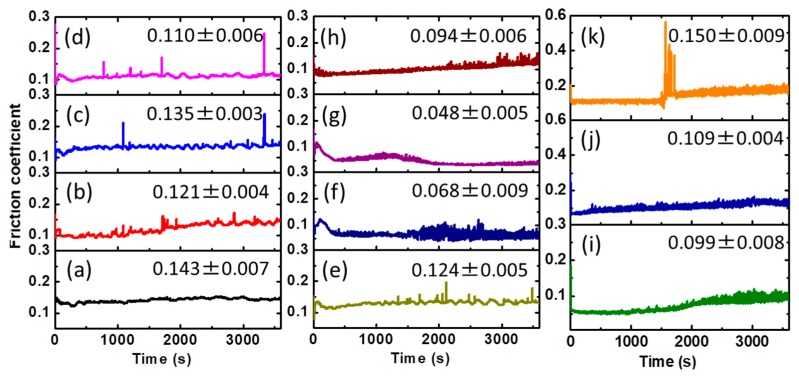
Friction coefficient while lubricating by EG/lignin lubricants. Load: 2.5 GPa, testing duration: 1 h, temperature: 25 °C. (**a**): EG; (**b**): EG-17BL; (**c**): EG-17SL; (**d**): EG-29BL; (**e**): EG-29SL; (**f**): EG-38BL; (**g**): EG-38SL; (**h**): EG-44BL; (**i**): EG-44SL; (**j**): EG-50BL, (**k**): EG-50SL. The value in the figure is the average friction coefficient of three friction tests.

Figure 4 shows the friction coefficient evolution with the presence of EG and EG/lignin lubricants under a pressure of 2.5 GPa. A relatively higher friction coefficient is observed by using pure EG as a lubricant. The addition of lignin to EG definitely helps to reduce the friction coefficient. However, it seems that the friction coefficient becomes unstable, as evidenced by the fluctuation during the friction process, even when the lignin loading goes up to 29 wt%. When increasing the lignin loading to 38 wt%, the friction coefficient can be successfully stabilized at 0.068 and 0.048, as shown in Figure 4g,h. A further lignin loading increase to 50 wt% leads to an increase of the friction coefficient. Taking the average friction coefficient of three friction tests as a measure, the friction coefficient can be reduced by 6.6–66.4% with the addition of 17–50 wt% SL or BL compared to pure EG (0.143). It is important to select a lubricant of appropriate viscosity to achieve optimum lubrication [40]. At a low viscosity range, the addition of lignin increases the viscosity and thus improves the lubrication. The enhanced viscosity could increase the thickness of lubricating films, effectively prevent direct metal/metal contact, and thus reduce the friction coefficient [41]. The further enhanced viscosity with a larger lignin faction would lead to high internal friction force and accumulate a large amount of friction heat, leading to a disturbed friction coefficient [21]. The lowest friction coefficient at 38% lignin loading could be attributed to the balance between the friction heat and viscosity. The average friction coefficient relationship between EG-SL and EG-BL varies at different lignin loadings. The average friction coefficient of EG-SL is larger than that of EG-BL at the majority of lignin loadings except 38%. At a low viscosity range, the larger viscosity of EG-17/29BL effectively prevents the metal/metal contact and leads to a lower friction coefficient as compared with EG-17/29SL. Meanwhile, at a high viscosity range, the enhanced viscosities of EG-38BL, EG-44SL, and EG-50SL could induce a higher internal friction force and thus lead to an increased friction coefficient as compared with EG-38SL and EG-44/50BL, respectively.

**Figure 5:**
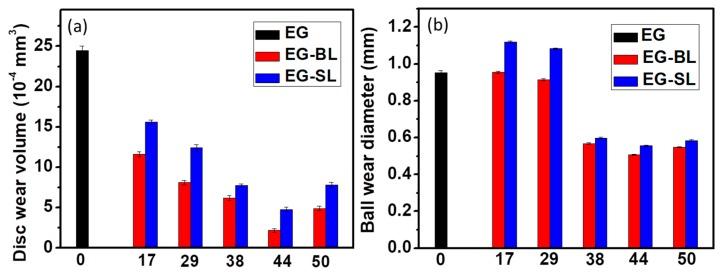
Disc wear volume (**a**) and ball wear diameter (**b**) lubricated by two EG-based lubricants. Load: 2.5 GPa, testing duration: 1 h, temperature: 25 °C.

Figure 5 shows the wear volume loss of the discs and wear diameter of the ball lubricated by various EG-based lubricants. The disc wear volume loss lubricated by EG/lignin is apparently lower than that lubricated by pure EG, which indicates the positive contribution of organosolv lignin in promoting the anti-wear properties of the lubricant. The disc wear volume loss decreases continuously with increasing the lignin loading, reaches the minimum value at 44 wt%, and goes up afterwards. Specifically, the wear volume of the disc by EG-44BL and EG-44SL is only 8.9% and 19.5% of pure EG. Moreover, the disc wear loss of EG-BL is lower than that of EG-SL at each lignin loading. For example, the disc wear loss of EG-44BL is only 45.7% of that lubricated by EG-44SL. 

The ball wear diameter is another important indicator of anti-wear properties. With a reduced disc wear volume achieved by adding 17 and 29 wt% lignin in EG, an enlarged ball wear diameter was surprisingly observed, see Figure 5b. The anti-wear properties are synergistically affected by the adhesion of lubricants on the friction surface and the mechanical strength of the lubrication film [42,43]. At low lignin loadings (17% and 29%), protecting films cannot be formed, which leads to an increased ball wear diameter. EG-38 SL/BL has a lower ball wear diameter than that of pure EG. The ball wear diameter continuously decreases in EG-44SL/BL and goes up afterwards. The ball diameter of EG-44BL and EG-44SL is only 53.1% and 58.3% of pure EG. It is similar to the disc wear volume, in that the ball wear diameter lubricated by EG-BL is lower than that of EG-SL. With an increase of lignin loading, the lubricating film can be strengthened to reduce the wear rate of metal/metal contact. With further increasing the lignin loading to 50 wt%, the large viscosity makes the lubricant gradually lose its good lubrication properties and thus increases the wear rate [41,44]. The optimal loading for the lowest wear volume was found at 44%.

**Figure 6:**
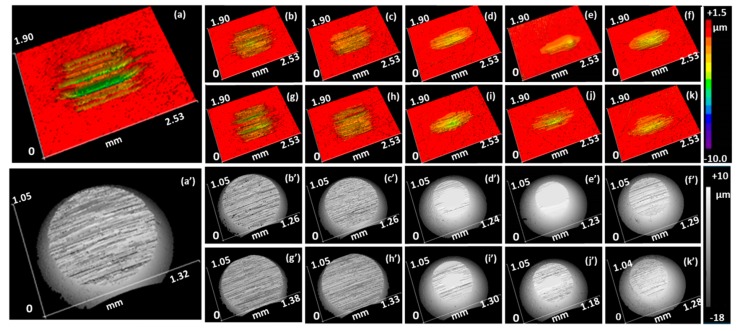
Three-dimensional (3D) disc and ball wear pictures while lubricating by lignin/EG lubricants. Load: 2.5 GPa, testing duration: 1 h, temperature: 25 °C. Disc wear images: (**a**): EG; (**b**): EG-17BL; (**c**): EG-29BL; (**d**): EG-38BL; (**e**): EG-44BL; (**f**): EG-50BL; (**g**): EG-17SL; (**h**): EG-29SL; (**i**): EG-38SL; (**j**): EG-44SL; (**k**): EG-50SL. Ball wear images: (**a**′): EG; (**b′**): EG-17BL; (**c′**): EG-29BL; (**d′**): EG-38BL; (**e′**): EG-44BL; (**f′**): EG-50BL; (**g′**): EG-17SL; (**h′**): EG-29SL; (**i′**): EG-38SL; (**j′**): EG-44SL; (**k′**): EG-50SL.

Figure 6 shows the three-dimensional (3D) surface profiles of the wear tracks on disc and ball after a friction test using different lubricants. The disc wear track with pure EG is obviously deeper and larger than that lubricated by EG/lignin lubricants. From the first to sixth columns at the same row in Figure 6, the smallest wear volume and ball diameter were obtained at 44% lignin loading. Comparing the 3D images from first/second or third/fourth rows of Figure 6, the EG-BL lubricants show relatively smaller and shallower wear tracks than the EG-SL lubricants, which further confirms the superior anti-wear properties of EG-BL. The better anti-wear properties of EG-BL over EG-SL are most likely attributed to the denser H-bonding sites in BL and its broader molecular weight distribution. Concerning the molecular structure, BL has more functional group sites to form stronger H-bonding with EG, increasing the strength of the lubrication film. The broader molecular weight distribution of BL would promote lignin adhesion on the metal surface, where the higher molecular weight lignin constructs a strong network to provide the mechanical strength of lubricating films, while lower molecular weight lignin fills in the free space on unoccupied surface to provide surface lubrication [45,46]. Therefore, BL with a broader molecular weight distribution shows a better adhesion on metal surfaces and exhibits excellent anti-wear properties.

## 3. Experimental Section

### 3.1. Materials

Ethylene glycol (anhydrous, 99.8%) was purchased from Sigma Aldrich (Saint Louis, MO, USA). Spruce and birch lignins were prepared by the organosolv pretreatment method from Norway spruce (*Picea abies*) and silver birch (*Betula pendula*) chips. The detailed preparation method is described in our previous publication [17]. All chemicals and materials were used as received without further treatment.

### 3.2. Preparation of Lubricants

Spruce organosolv lignin of different weight fractions (17, 29, 38, 44, and 50 wt%) was added to EG at room temperature and then heated at 140 °C in a 150W microwave processor (Discover SP, CEM, Matthews, NC, USA) with magnetic stirring for 10 min. After the microwave processing, homogeneous solutions were formed and denoted as EG-17SL, EG-29SL, EG-38SL EG-44SL, and EG-50SL. The same procedure was applied to dissolve birch organosolv lignin in EG and the prepared mixtures were named EG-17BL, EG-29BL, EG-38BL EG-44BL, and EG-50BL, respectively.

### 3.3. Characterization

The thermal stability of pure EG and their mixtures with spruce or birch lignin was determined by thermogravimetric analysis (TGA, TA instrument Q500) in N_2_ atmosphere from 20 to 600 °C with a heating rate of 10 °C/min. Fourier transform infrared-attenuated total reflection (FT-IR-ATR) spectra were recorded with a Thermo Scientific Nicolet 380 series spectrometer (Thermo Fisher Scientific, Waltham, MA, USA). The lubricant viscosity was reported within a shear rate range of 1~100 s^−1^ using a Bohlin CVO 100 rheometer (Malvern Instruments, Malvern, UK) at 25 °C. A cone-on-plate geometry was used with a 1° cone angle and 20-mm cone diameter. The lower plate had a diameter of 60 mm. 

An Optimol SRV-III oscillating friction and wear tester was used to evaluate the tribological properties of the prepared lubricants based on ASTM D 6425 protocol. During the test, the upper steel ball (52,100 bearing steel, diameter: 10 mm, surface roughness (*R_a_*): 20 nm) slides under reciprocating motion against a stationary steel disc (100CR6 ESU-hardened, *Ø* 24 mm × 7.9 mm, surface roughness (*R_a_*): 120 nm). The disc was supplied by Optimol Instruments Prüftechnik GmbH, Germany. The ball was provided by SKF, Göteborg, Sweden. Before each test, the device and sample were cleaned with acetone and ethanol, followed by a uniform application of 0.5 mL lubricant on the steel disc using a glass rod. All tests were conducted under a load of 150 N (2.5 GPa Maximum Hertzian pressure) at 25 °C, a sliding frequency of 50 Hz, and an amplitude of 1.0 mm. The friction coefficient curves were recorded automatically with a data acquisition system linked to the SRV-III tester. After the tests, the wear volumes of the lower discs and wear diameters of the higher balls were determined using an optical profiling system (Zygo 7300). Three duplicate friction and wear tests were carried out to minimize the experimental error.

## 4. Conclusions

Organosolv lignin dissolution was dissolved in EG up to 50 wt%. The addition of organosolv lignin in EG results in outstanding lubricating properties. The enhanced anti-wear property of the EG/lignin system could be attributed to its excellent adhesion ability on the metal surface and superior lubrication film strength. Specifically, the wear volume of the disc lubricated by EG-44BL is only 8.9% of that lubricated by pure EG. The viscosity difference between EG-BL and EG-SL at different lignin loadings depends on the balance between the thickening effect of lignin itself and the H-bonding between lignin and EG. The lubrication property of EG-BL exceeds that of EG-SL, which could be attributed to the denser H-bonding sites in the molecular structure of BL as well as its broader molecular weight distribution. This work offers a new avenue of utilizing bio-products from different sources in advanced tribological lubrication systems. 

## Figures and Tables

**Table 1:** Average molecular weight of lignin extracted from birch (BL) and spruce biomass (SL). Reprinted with permission from [17]. Copyright (2016) American Chemical Society.

	BL	SL
*M*_w_ (Da)	1855	2226
*M_n_* (Da)	587	846
PDI	3.2	2.6
